# A resource-saving collective approach to biomedical semantic role labeling

**DOI:** 10.1186/1471-2105-15-160

**Published:** 2014-05-27

**Authors:** Richard Tzong-Han Tsai, Po-Ting Lai

**Affiliations:** 1 Department of Computer Science and Information Engineering, National Central University, Taoyuan, Taiwan, Republic of China; 2 Department of Computer Science, National Tsing-Hua University, HsinChu, Taiwan, Republic of China; 3 Institute of Information Science, Academia Sinica, Taipei, Taiwan, Republic of China

## Abstract

**Background:**

Biomedical semantic role labeling (BioSRL) is a natural language processing technique that identifies the semantic roles of the words or phrases in sentences describing biological processes and expresses them as predicate-argument structures (PAS’s). Currently, a major problem of BioSRL is that most systems label every node in a full parse tree independently; however, some nodes always exhibit dependency. In general SRL, collective approaches based on the Markov logic network (MLN) model have been successful in dealing with this problem. However, in BioSRL such an approach has not been attempted because it would require more training data to recognize the more specialized and diverse terms found in biomedical literature, increasing training time and computational complexity.

**Results:**

We first constructed a collective BioSRL system based on MLN. This system, called collective BIOSMILE (CBIOSMILE), is trained on the BioProp corpus. To reduce the resources used in BioSRL training, we employ a tree-pruning filter to remove unlikely nodes from the parse tree and four argument candidate identifiers to retain candidate nodes in the tree. Nodes not recognized by any candidate identifier are discarded. The pruned annotated parse trees are used to train a resource-saving MLN-based system, which is referred to as resource-saving collective BIOSMILE (RCBIOSMILE). Our experimental results show that our proposed CBIOSMILE system outperforms BIOSMILE, which is the top BioSRL system. Furthermore, our proposed RCBIOSMILE maintains the same level of accuracy as CBIOSMILE using 92% less memory and 57% less training time.

**Conclusions:**

This greatly improved efficiency makes RCBIOSMILE potentially suitable for training on much larger BioSRL corpora over more biomedical domains. Compared to real-world biomedical corpora, BioProp is relatively small, containing only 445 MEDLINE abstracts and 30 event triggers. It is not large enough for practical applications, such as pathway construction. We consider it of primary importance to pursue SRL training on large corpora in the future.

## Background

### Biomedical semantic role labeling (BioSRL)

**Figure 1:**
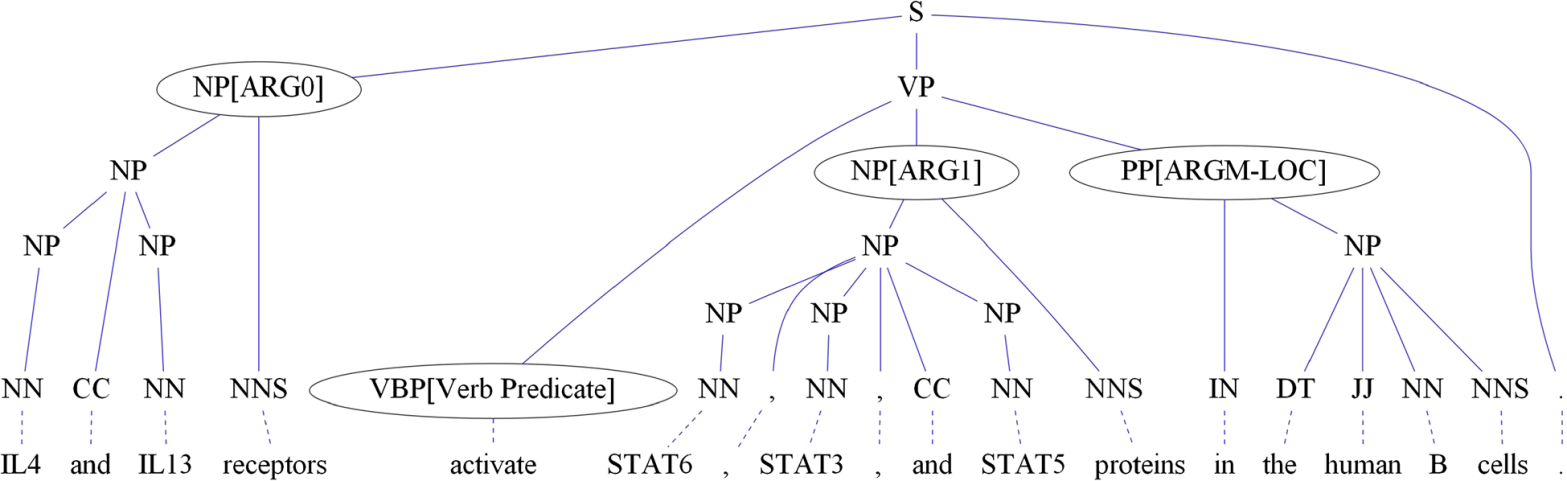
An example parse tree annotated with semantic roles.

Biomedical semantic role labeling (BioSRL) is an important natural language processing technique for life scientists who are interested in uncovering information related to biological processes within literature. In BioSRL, sentences are represented by one or more predicate argument structures (PASs), also known as propositions [1]. Each PAS is composed of a predicate (a verb) and several arguments (e.g., noun phrases) that have different semantic roles, including main arguments such as agent and patient, as well as adjunct arguments, such as time, manner, and location. Here, the term argument refers to a syntactic constituent of the sentence related to the predicate, and the term semantic role refers to the semantic relationship between a predicate and an argument of a sentence. For example, in Figure 1, the sentence "IL4 and IL13 receptors activate STAT6, STAT3, and STAT5 proteins in the human B cells," describes a molecular activation process. It can be represented by a PAS in which "activate" is the predicate, "IL4 and IL13 receptors" and "STAT6, STAT3, and STAT5 proteins" comprise ARG0 (agent) and ARG1 (patient), respectively, with "in the human B cells" as the location. Thus, the agent, patient, and location are the arguments of the predicate.

Given a sentence, the SRL task executes two steps: predicate identification and argument recognition. The first step can be achieved by using a part-of-speech (POS) tagger with some filtering rules. Then, the second step recognizes all arguments, including grouping words into arguments and classifying the arguments into semantic role categories. Some studies refer to these two sub-steps as argument identification and argument classification, respectively [2,3]. In the second step, it is often difficult to determine the word boundaries and semantic roles of an argument as they depend on many factors, such as the argument's position, the predicate's voice (active or passive) and the sense (usage). The second step can be formulated as a sentence tagging problem. A sentence can be represented by a sequence of words, a sequence of phrases, or a parse tree; the basic units of a sentence are words, phrases, and constituents (a node on a full parse tree) arranged in the above representations, respectively. Hacioglu et al. [4] showed that tagging phrase-by-phrase (P-by-P) is better than word-by-word (W-by-W). However, Punyakanok et al. [3] showed that constituent-by-constituent (C-by-C, or node-by-node) tagging is better than P-by-P. Based on Punyakanok et al.’s findings, BIOSMILE [5] also adopted C-by-C tagging for BioSRL and achieved an accuracy close to that of top general SRL systems.

C-by-C approaches can be called “discrete approaches” because they do not consider dependencies among constituents/nodes. For example, a parent node and any of its children nodes cannot both be labeled with semantic roles simultaneously. In SRL, collective approaches which label several or all nodes simultaneously have been proposed and outperform discrete approaches [6]. The Markov logic network (MLN) model is a good representative collective approach. It offers the flexibility to model dependencies with first-order-logic formulae.

In this paper, we explore the collective approach to BioSRL by building an MLN-based system. Despite the convenience of modeling dependencies and the high accuracy of MLN, we have observed that it requires more memory and longer training times on a large corpus. This is an obstacle to applying MLN to BioSRL, which requires a large amount of training data to cover the wide variety of specialized biomedical subdomains. To reduce the resources used in BioSRL training, we employ a tree-pruning filter to remove unlikely nodes from the parse tree and four argument candidate identifiers to retain candidate nodes in the tree. Nodes not recognized by any candidate identifier are discarded. The pruned annotated parse trees are used to train a resource-saving MLN-based system, which is referred to as resource-saving collective BIOSMILE (RCBIOSMILE).

## Methods

**Figure 2:**
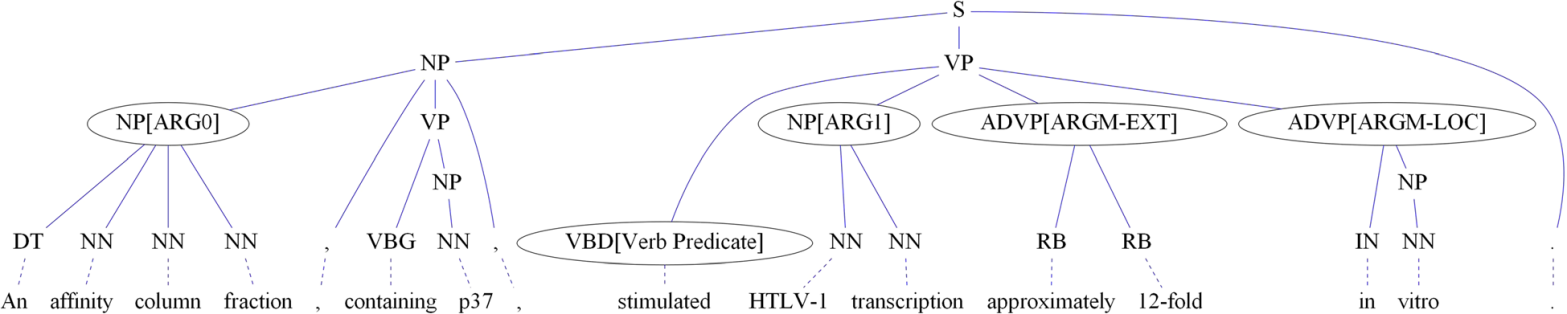
An example parse tree annotated with semantic roles.

In this section, we will firstly describe our main contribution: resource-saving preprocessing. Then, we will illustrate the Markov-logic-network-based collective learning approach. Before entering into the explanation of our methods, we define the terms used in this section. Given a sentence *s* and its full parse tree *p*, every node *n*_*i*_ in *p* corresponds to some substring of *s*, referred to as *sub*_*i*_. For the convenience of explanation, *sub*_*i*_ is referred to as *n*_*i*_’s span. Take the sentence in Figure 2 for example, the node NP[ARG1]’s span is “HTLV-1 transcription”. ARG1 is this node’s semantic role or argument type. All nodes with semantic roles are referred to as “argument nodes”. In SRL, predicate means the verb while in MLN, predicate represents relationships among objects in the first order logic. To avoid the ambiguity between the predicates in FOL and SRL, we follow Punyakanok et al. [3] to refer to the predicates in SRL as “verb predicate (VP)” afterwards.

### Resource-saving preprocessing

To reduce the resources used in BioSRL training, we employ a tree-pruning filter to remove unlikely nodes from the parse tree and four argument candidate identifiers to retain candidate nodes in the tree. Nodes not recognized by any candidate identifier are discarded. In the following subsections, we describe these components.

### Tree pruning filter (TPF)

**Figure 3:**
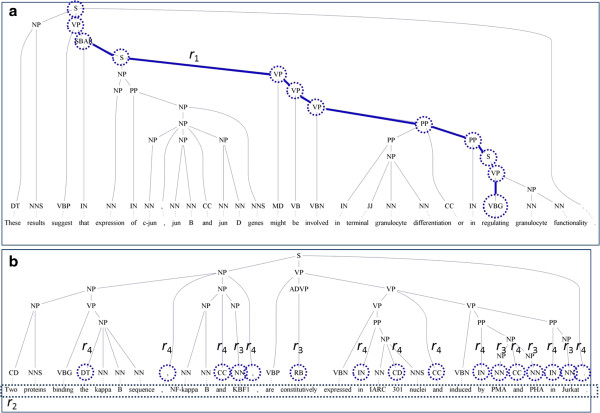
**Examples of tree pruning. a**. Pruning nodes using pruning rule 1. **b**. Pruning nodes using rules 2, 3, and 4.

We employ four rules to prune nodes unlikely to be arguments. The first rule is based on the intuition that if a node’s span overlaps with the verb predicate, the node is unlikely to have a semantic role. Such nodes are located in the same path as the node whose span is the verb predicate and must be removed. An example is shown in Figure 3a. Figure 3b shows the application of rules 2, 3, and 4. The second rule *r*_*2*_ is to remove all leaf nodes, which are enclosed in a dotted box. The third rule *r*_3_ is to remove all nodes without any siblings. These nodes are enclosed in dotted circles and labeled with “*r*_3_”. The fourth rule *r*_4_ is to remove all nodes whose spans are stop-words. These nodes are enclosed in dotted circles and labeled with “*r*_4_”.

### Association rule candidate identifier (ARCI)

Rules containing several predicates are effective for identifying argument candidates. For example, if a node *i* starts with “in” and ends with “cell”, then it is very likely to be a location argument (ARGM-LOC). This rule can be translated into the following first-order logic formula:

**Rule1:***firstword* (*i*, " in ") ∧ *lastword* (*i*, " cell ") ⇒ *role* (*p*, *i*, " ARGM ‒ LOC ")

We can see that this rule is composed of three predicates: *firstword* (*i*, "in"), *lastword* (*i*, "cell"), and *role* (*p*, *i*, "ARGM - LOC").

However, compiling the set of rules is labor-intensive. To automatically generate a rule, the main task is to decide which predicates must be included. We select several basic predicate types listed as follows.

*The pool of predicates*

 Node type

 First word and last word stem

 POS of the first word and last word

 POS of the verb predicate

 Voice of the verb predicate

 Before or after the verb predicate

 Semantic role

 The verb predicate

 Syntactic path from the verb predicate

These features are selected from basic features used in the BIOSMILE [5] system. We formulate the rule-generation problem as a problem of association rule mining [7].

**Figure 4:**
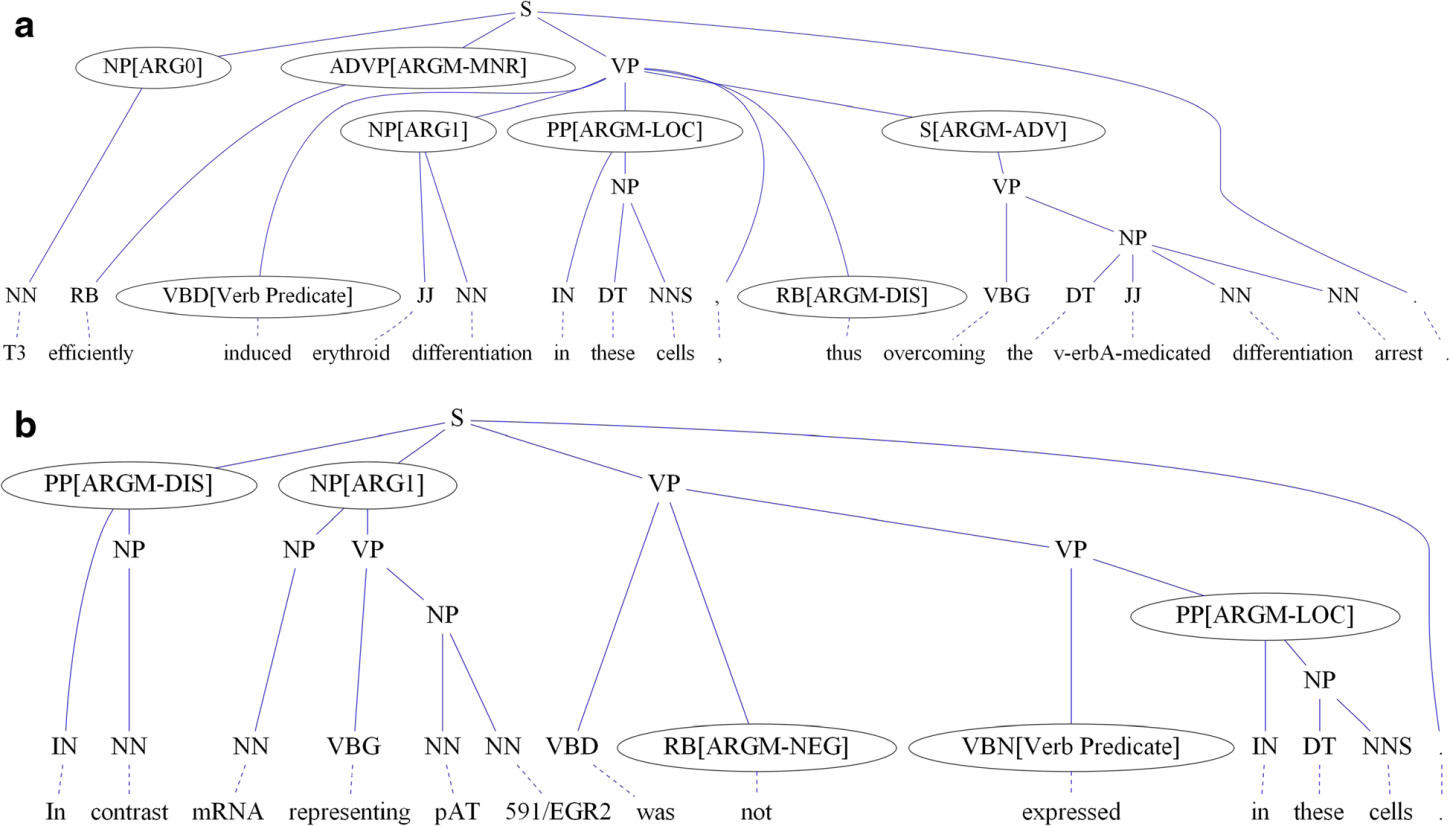
**Examples of association rule mining. a**. T3 efficiently induced erythroid differentiation in these cells, thus overcoming the v-erbA-mediated differentiation arrest. **b**. In contract mRNA representing pAT 591/EGR2 was not expressed in these cells.

To formulate the rule generation problem as an association-rule-mining task, it is necessary to define four things including *item*, *transaction*, *support* and *confidence*. An *item* is a predicate appearing in a rule, such as the predicates *firstword* (*i*, "in"), *lastword* (*i*, "cell"), and *role* (*p*, *i*, "ARGM - LOC") from the above example. To acquire *I*, the set of all items, we process the training set and extract predicates from arguments. A *transaction* is a collection of items. In this work, all arguments (nodes with semantic roles) in the training set are treated as transactions. For instance, the two sentences shown in Figure 4 can be transformed into the transactions shown in ‘The transactions extracted from the sentences in Figure 4. The full name of all abbreviations can be found in Table 1’. The *support* supp(*X*) of an itemset *X* is defined as the proportion of transactions in the data set which contain *X*. The *confidence* of a rule *X* ⇒ *Y* is defined as conf(*X* ⇒ *Y*) = supp(*X* ∩ *Y*)/supp(*X*). For example, **Rule 1** has a confidence of 0.021/0.022 in the database, which means that 95% of the transactions that follow the rule are correct. Using the apriori algorithm [8], rules such as

**Table 1:** The features used in BIOSMILE

**Basic features**	• Verb predicate – The verb predicate lemma
	• Path – The syntactic path through the parse tree from the constituent being classified to the verb predicate
	• Constituent type (CT)
	• Position – Whether the phrase is located before or after the verb predicate
	• Voice – passive if the verb predicate has a POS tag VBN, and its chunk is not a VP, or it is preceded by a form of "to be" or "to get" within its chunk; otherwise, it is active
	• Head word – Calculated using the head word table described by Collins (1999)
	• Head POS – The POS of the Head Word
	• Sub-categorization – The phrase structure rule that expands the predicate's parent node in the parse tree
	• First and last Word (FW and LW) and their POS tags
	• Level – The level in the parse tree
**Verb predicate features**	• Verb predicate's verb class
	• Verb predicate POS tag
	• Verb predicate frequency
	• Verb predicate's context POS
	• Number of verb predicates
**Full parsing features**	• Parent, left sibling, and right sibling paths, constituent types, positions, head words, and head POS tags
	• Head of Prepositional Phrase (PP) parent – If the parent is a PP, then the head of this PP is also used as a feature
**Combination features**	• Verb predicate distance combination
	• Verb predicate phrase type combination
	• Head word and verb predicate combination
	• Voice position combination
**Others**	• Syntactic frame of verb predicate/NP
	• Headword suffixes of lengths 2, 3, and 4
	• Number of words in the phrase
	• Context words & POS tags

$$\begin{array}{l} \mathrm{event}\_\mathrm{trigger}\;\left(p\right)\wedge \mathrm{node}\_\mathrm{type}\;(i,"\mathrm{PP}")\wedge \mathrm{firstword}\;(i,"\mathrm{in}")\wedge \\ \mathrm{lastword}\;\left(i,"\mathrm{cells}"\right)\wedge \mathrm{lastword}\_\mathrm{POS}\;(i,"\mathrm{NNS}")\\ \Rightarrow \mathrm{candidate}\_\mathrm{role}\;\left(p,i,"\mathrm{ARGM}‒\mathrm{LOC}"\right) \end{array}$$

can be generated.

The generated association rules are employed to identify argument candidates. Nodes not matching any rule are discarded.

***The transactions extracted from the sentences in Figure*** 4 ***. The full name of all abbreviations can be found in Table*** 1

FW(T3), LW(T3), CT(NP), PATH(NP > S < VP < VBD), verb_predicate(induce), ROLE(ARG0)

FW(efficiently), LW(efficiently), CT(ADVP), PATH(ADVP > S < VP < VBD), verb_predicate(induce), ROLE(ARGM-MNR)

FW(erythroid), LW(differentiation), CT(NP), PATH(VBD > VP < NP), verb_predicate(induce), ROLE(ARG1)

FW(in), LW(cell), CT(PP), PATH(VBD > VP > S < PP), verb_predicate(induce), ROLE(ARGM-LOC)

FW(thus), LW(thus), CT(RB), PATH(VBD > VP < RB), verb_predicate(induce), ROLE(ARGM-DIS)

FW(overcoming), LW(arrest), CT(S), PATH(VBD > VP < S), verb_predicate(induce), ROLE(ARGM-ADV)

FW(in), LW(contrast), CT(PP), PATH(PP > S < VP < VP < VBN), verb_predicate(express), ROLE(ARGM-DIS)

FW(mRNA), LW(591/egr2), CT(NP), PATH(NP > S < VP < VBN), verb_predicate(express), ROLE(ARG1)

FW(not), LW(not), CT(RB), PATH(RB > VP < VBD), verb_predicate(express), ROLE(ARGM-NEG)

FW(in), LW(cell), CT(PP), PATH(VBD > VP < S < PP), verb_predicate(express), ROLE(ARGM-LOC)

### Word-based candidate identifier (WCI)

Some types of arguments can be identified by checking if they exactly match specific words with other conditions. We compile lists of words corresponding to three types of arguments. These argument types are:

***Discourse Argument *** **(ARGM-DIS):** Discourse arguments connect sentences to preceding sentences. If a node’s span can be found in the word list for ARGM-DIS, the node is regarded as an ARGM-DIS candidate. The word lists for ARGM-DIS, ARGM-MOD and ARGM-NEG are shown in Table 2.

**Table 2:** The word list for identifying ARGM-DIS, ARGM-MOD, ARGM-NEG

**Type**	**Word list**
ARGM-DIS	Additionally, also, altogether, as well as, but also, by contrast, conversely, even, finally, further, furthermore, hence, however, importantly, in addition, in conclusion, in contrast, in fact, in parallel, in part, in particular, in sum, in summary, in this regard, in turn, indeed, instead, interestingly, likewise, moreover, nevertheless, no longer, nonetheless, not only, on the contrary, on the other hand, probably, rather, still, surprisingly, then, thereby, therefore, thus, whereas
ARGM-NEG	Not, n’t, never, no longer
ARGM-MOD	Can, could, may, might, shall, should, would, will, going (to), have (to), use (to)

***Modal Argument *** **(ARGM-MOD) and *Negation Argument *** **(ARGM-NEG):** If a node’s span appears right before a verb predicate and can be found in the word list for either ARGM-MOD or ARGM-NEG, it is regarded as an ARGM-MOD or ARGM-NEG candidate, respectively.

### Pattern-based candidate identifier (PCI)

***Extent Marker*** **(ARGM-EXT):** The extent marker indicates the amount of change caused by an action, such as “approximately 12-fold” in Figure 2. We have observed that extent markers are usually siblings of the verb-predicate node (the VBD[Verb Predicate] node in Figure 2). For each sibling *sib*, the identifier checks whether the subtree with root *sib* has any nodes whose spans match the extent marker pattern (shown in Table 3). Matching nodes are regarded as **ARGM-EXT** candidates.

**Table 3:** Extent and temporal marker patterns

**Pattern type**	**Regular expression**
Extent	\b(\d + %|fold|extent|(greater|less) than \d+)\b
Temporal	\b(year|month|week|day|hour|minute|min|second|sec|(\d + |one|several)[\-]?(wk|hr|h))s?\b

***Temporal Marker *** **(ARGM-TMP):** The temporal marker indicates when an action takes place. Like extent markers, temporal markers are usually siblings of the verb-predicate node and are identified in the same manner. The identifier finds **ARGM-TMP** candidates by checking whether the subtrees of verb-predicate node with root *sib* have any nodes whose spans match the temporal marker pattern. In addition, temporal markers sometimes appear at the beginning of a sentence. Therefore, the identifier also checks if any nodes whose spans start with the first word of a sentence match the temporal marker pattern (shown in Table 3). Such nodes are also considered **ARGM-TMP** candidates.

### Parse-tree-based candidate identifier (PTCI)

If a node *n* is not identified as a candidate by the above components, this module will check if the path from the verb-predicate node to *n* is equal to any path from the verb-predicate node to an argument node *m* in the training set. If yes, *n* will be treated as a candidate of *m*’s argument type.

### Markov logic

#### First-order logic

MLN combines first order logic (FOL) and Markov networks. In FOL, formulae consist of four types of symbols: constants, variables, functions, and predicates. *Constant* symbols represent objects in a specific domain (e.g., Annie, Bob, Cathy, etc.). *Variable* symbols range over the objects in the domain. *Function* symbols (e.g. MotherOf) represent mappings from tuples of objects to objects. *Predicates* represent relationships among objects (e.g. Friends), or attributes of objects (e.g. Smokes). Constants and variables may belong to specific types. An *atom* is a predicate symbol applied to a list of arguments, which may be constants or variables. A *ground atom* is an atom whose arguments are all constants. A *world* is an assignment of truth values to all possible ground atoms. A *knowledge base* (KB) is a partial specification of a world; each atom in it is true, false, or unknown.

#### Markov networks

A Markov network represents the joint distribution of a set of variables *X* = {*X*_1_, …, *X*_*n*_} ∈ X as a product of factors: $P\left(X=x\right)=\frac{1}{Z}\prod_{k}f_{k}\left(x_{k}\right)$, where each factor *f*_*k*_ is a non-negative function of a subset of the variables *x*_*k*_, and *Z* is the normalization constant. The distribution is usually equivalently represented as a log-linear form: $P\left(X=x\right)=\frac{1}{Z}\exp\left(\sum_{i}w_{i}g_{i}\left(x\right)\right)$, where the features *g*_*i*_(*x*) are arbitrary functions of (a subset of) the variables’ states.

#### Markov logic networks

An MLN is a set of weighted first-order formulae. Together with a set of constants representing objects in the domain, it defines a Markov network with one variable per ground atom and one feature per ground formula. The probability distribution over possible worlds is given by $P\left(X=x\right)=\frac{1}{Z}\exp\left(\sum_{i\in F}\sum_{j\in G_{i}}w_{j}g_{j}\left(x\right)\right)$ where *Z* is the partition function, ***F*** is the set of all first-order formulae in the MLN, ***G***_***i***_ is the set of groundings of the *i*-th first-order formula, and *g*_*j*_(*x*) = 1 if the *j*-th ground formula is true, and *g*_*j*_(*x*) = 0 otherwise. Markov logic enables us to compactly represent complex models in non-i.i.d. domains. General algorithms for inference and learning in Markov logic are discussed in Richardson and Domingos [9]. We use the1-best MIRA online learning method [10] for learning weights and employ cutting plane inference [11] with integer linear programming as its base solver for inference at test time as well as during the MIRA online learning process. As aforementioned, to avoid the ambiguity between the predicates in FOL and SRL, we refer to SRL predicates in as “verb predicate”.

### Formulae

#### Local formulae (L)

As shown in Table 1, local formulae are derived from the features used in the SRL systems [2,12-14] based on the maximum entropy (ME) model and support vector machine (SVM) model. We used these features in BIOSMILE [5], and we have transformed them into formulae here to employ them in our MLN model.

A local formula consists of two observed predicates, one corresponding to the verb predicate and the other one denoting a feature of a node. For example, the headword feature can be expressed in FOL as

$$\mathrm{candidate}\_\mathrm{role}\left(\;p,i,+r\right)\wedge \mathrm{headword}\left(\;i,+w\right)\;\Rightarrow \mathrm{role}\left(\;p,i,+r\;\right)$$

where *w* is the headword of the node *i*. If the “+” symbol appears before a variable, it indicates that each different value of the variable has its own weight.

#### Collective formulae (C)

Collective classification is a methodology that simultaneously classifies related instances. It can improve classification accuracy over non-collective methods when instances are interrelated [15-17]. MLN performs well in many collective classification tasks such as entity linking [18-20], coreference resolution [21,22] and biomedical event extraction [23]. In node-by-node SRL, related instances are nodes having linguistic dependencies.

There are two main types of linguistic dependencies in SRL: tree dependency and path dependency. They have been shown to be effective in improving the consistency of SRL results [3]. Nodes with tree dependencies and path dependencies can be treated as tree collectives and path collectives, respectively. In MLN-based SRL, collectives can be implemented with collective formulae that model dependencies among nodes.

Given a sentence *sen* and a verb predicate *p*, a tree collective is composed of all nodes in *sen*’s parse tree. In this tree collective, there is a constraint that each core semantic role of *p* can only be assigned to one node, which can be expressed in the following formula:

$$\mathrm{verb}\_\mathrm{predicate}\left(p\right)\wedge \mathrm{core}\_\arg\left(+r\right)\Rightarrow \left|\mathrm{role}\left(p,i,+r\right)\right|\leq 1$$

In addition, a path collective is composed of all nodes in a path. Spans of nodes in the same path collective overlap. Therefore, only one node in a path can play a semantic role. This dependency can be formulated as follows:

$$\mathrm{overlap}\left(i,j\right)\wedge \mathrm{role}\left(p,i,r_{1}\right)\Rightarrow \left|\mathrm{role}\left(p,j,r_{2}\right)\right|=0$$

#### Candidate identification formulae (CI)

In our resource-saving preprocessing step, candidate identifiers recognize the most likely semantic roles for each node. The information can be transformed into formulae to improve the accuracy of MLN inference. For a node *i* retained as a candidate node by our resource-saving preprocessing, an observed predicate *candidate_role*(*p*, *i*, *r*) is added to our MLN-based inference system.

## Results

### Dataset

We use BioProp [24] as our evaluation dataset. BioProp is a semantic role labeling corpus which contains 445 biomedical abstracts containing 1,982 PAS’s labeled with the 30 most common biomedical verb predicates and their semantic roles. Table 4 shows the statistics of the BioProp corpus.

**Table 4:** The statistics of the BioProp corpus

**Role**	**Number**
Core argument types	11
Adjunctive argument types	21
**Feature**	**Number**
Constituent types	17
Unique words	5258
Part-of-speech	34
**Other**	**Number**
Verb predicate types	30
Abstracts with PAS’s	445
Sentences with PAS’s	1622
Propositions	1962

Core arguments such as ARGX, R-ARGX and C-ARGX play the main semantic roles in a PAS. ARGX (ARG0–ARG5, ARGX) are the most necessary arguments of a given verb predicate. C-ARGX is used to represent multi-node arguments. A node labelled C-ARGX is assumed to be a continuation of the closest node to the left labelled ARGX. A node labelled R-ARGX is assumed to be a relative pronoun of the closest node to the left labelled ARGX. Adjunctive arguments (ARGM-X) play the semantic roles of location, manner, time, or extent in a PAS.

### Evaluation metric

The argument-wide results are given as F-score using the CoNLL-05 [25] evaluation script and defined as $F=\frac{2\times P\times R}{P+R}$, where *P* denotes the precision and *R* denotes the recall. The formulae for calculating *P* and *R* are as follows:

$$P=\frac{\mathrm{the}\;\mathrm{number}\;\mathrm{of}\;\mathrm{correctly}\;\mathrm{recognized}\;\mathrm{arguments}}{\mathrm{the}\;\mathrm{number}\;\mathrm{of}\;\mathrm{recognized}\;\mathrm{arguments}}$$

$$R=\frac{\mathrm{the}\;\mathrm{number}\;\mathrm{of}\;\mathrm{correctly}\;\mathrm{recognized}\;\mathrm{arguments}}{\mathrm{the}\;\mathrm{number}\;\mathrm{of}\;\mathrm{arguments}}$$

Furthermore, we also evaluate the results in terms of the PAS-wide F-score (*F*_*PAS*_), which is defined as $F_{\mathrm{PAS}}=\frac{2\times P_{\mathrm{PAS}}\times R_{\mathrm{PAS}}}{P_{\mathrm{PAS}}+R_{\mathrm{PAS}}}$. The formulae for calculating *P*_*PAS*_ and *R*_*PAS*_ are as follows:

PPAS=thenumberofcorrectlyrecognizedPAS'sthenumberofrecognizedPAS's

RPAS=thenumberofcorrectlyrecognizedPAS'sthenumberofPAS's

### *t*-test

In order to evaluate our performance under an unbiased circumstance, we apply a two-sample paired *t*-test, which is defined as follows:

The null hypothesis, which states that there is no difference between the two configurations A and B, is given as

$$H_{0}:\mu_{A}=\mu_{B}$$

where *μ*_*A*_ is the true mean F-score of configuration A and *μ*_*B*_ is the mean of the configuration B, while the alternative hypothesis is

$$H_{1}:\mu_{A}>\mu_{B}$$

A two-sample paired *t*-test is applied since we assume the samples are independent. As the number of samples is large and the samples’ standard deviations are known, the following two-sample *t*-test can be administered:

$$t=\frac{\left(\bar{X_{A}}-\bar{X_{B}}\right)}{\sqrt{\frac{S_{A}^{2}}{n_{A}}+\frac{S_{B}^{2}}{n_{B}}}}$$

If the resulting *t*-score is equal to or less than 1.67 with a degree of freedom of 29 and a statistical significance level of 95%, the null hypothesis is accepted; otherwise it is rejected.

To retrieve the average F-scores and their deviations required for the *t*-test, we randomly sampled thirty training sets (*g*_1_, …,*g*_30_) and thirty test sets (*d*_1_, …, *d*_30_) from the 445 abstracts. Each training set and test set contains 365 and 89 abstracts, respectively. We trained the model on *g*_i_ and tested it on *d*_i_. Afterwards, we summed the scores for all thirty test sets and calculated the averages for performance comparison.

### Configuration settings

We construct three configurations of our system for comparison. The BIOSMILE configuration uses the local formulae (L), which is equivalent to our previous work. The CBIOSMILE configuration uses both the local (L) and collective formulae (C). In the RCBIOSMILE configuration, we firstly employ our proposed resource-saving preprossing (RP) step. Then, the local (L), collective (C), and candidate identification formulae (CI) are all used. The settings of these three configurations are shown in Table 5.

**Table 5:** Configuration settings and argument-wide SRL performance

**Configuration**	**Feature/Model/Preprocessing**					**ARGX**				**ARGM**				**Overall ARG**			
	**RP**	**L**	**MLN**	**C**	**CI**	**P**	**R**	**F**	**ΔF**	**P**	**R**	**F**	**ΔF**	**P**	**R**	**F**	**ΔF**
BIOSMILE		✓	✓	✓		91.59	85.48	88.43	-	81.36	67.50	73.79	-	88.72	80.00	84.14	-
CBIOSMILE		✓	✓	✓		90.44	89.19	89.81	+1.38^*^	81.12	67.45	73.66	−0.13	87.93	82.57	85.16	+1.02^*^
RCBIOSMILE: TPF	TPF	✓	✓	✓		90.47	89.22	89.84	+1.41*	81.16	65.99	72.79	−1	88.00	82.14	84.97	+0.83
RCBIOSMILE: ARCI	ARCI	✓	✓	✓	ARCI	90.76	88.55	89.64	+1.21*	82.18	66.65	73.6	−0.19	86.6	81.87	85.04	+0.9*
RCBIOSMILE: WCI	WCI	✓	✓	✓	WCI	90.4	89.24	89.85	+1.24*	80.77	68.34	74.04	+0.25	87.77	82.92	85.27	+1.13*
RCBIOSMILE: PCI	PCI	✓	✓	✓	PCI	90.38	89.24	89.8	+1.37*	77.59	70.91	74.1	+0.31	86.68	83.65	85.14	+1*
RCBIOSMILE: PTCI	PTCI	✓	✓	✓	PTCI	90.4	89.08	89.74	+1.31*	77.29	70.98	74	+0.21	86.6	83.56	85.05	+0.91*
RCBIOSMILE	All	✓	✓	✓	All	90.86	89.34	90.10	+1.67*	78.14	70.14	73.93	+0.14	87.23	83.49	85.32	+1.18*

*significantly outperforms BIOSMILE.

### Extraction performance

Table 5 shows the argument-wide performance of all configurations of our system on the CoNLL evaluation metrics, which measure whether each argument is independently correct. Table 6 shows configuration performance on PAS-wide evaluation metrics, in which a PAS is regarded as successfully extracted only if all its member arguments are correctly extracted. We use ‘*’ to indicate that the configuration shows a statistically significant improvement over BIOSMILE. CBIOSMILE is BIOSMILE with integrated collective learning. Table 5 shows that, across all arguments, CBIOSMILE outperforms BIOSMILE by 1.02% on average in terms of F-score. RCBIOSMILE boosts the improvement to 1.18%.

**Table 6:** PAS-wide SRL performance

**Configuration**	**ARGX**				**Overall ARG**			
	**P**	**R**	**F**	**ΔF**	**P**	**R**	**F**	**ΔF**
1. BIOSMILE	73.35	71.95	72.64	-	53.84	53.58	53.71	-
2. CBIOSMILE	79.43	79.33	79.38	+6.74^*^	58.31	58.31	58.31	+4.60^*^
3. RCBIOSMILE	80.01	79.57	**79.79**	+7.15^*^	59.53	59.46	**59.49**	+4.78^*^

In PAS-wide evaluation (Table 6), CBIOSMILE shows an improvement of 4.60% (F-score) over BIOSMILE, and an even larger advantage can be observed on core arguments (6.74%). RCBIOSMILE enlarges the improvement to 4.78% (F-score) over BIOSMILE, and an even larger advantage can be observed on core arguments (7.15%).

### Resource savings

We first examine the effects of tree pruning filters. On average, rules 1,2,3, and 4 removes 10%, 36%, 5%, and 13% of all nodes, respectively. Applying all four tree pruning rules can filter 43% of all nodes.

Table 7 displays time and memory costs. We compare each configuration’s training time per iteration and test time per instance. Compared to CBIOSMILE, which has similar performance (Table 6), RCBIOSMILE requires 92% less memory and 57% less training time, which are dramatic savings. We believe that RCBIOSMILE could be further improved by adding new SRL patterns written manually by biological experts.

**Table 7:** The cost of time and memory

**Configuration**	**Training time (per iter.)**	**Test time (per inst.)**	**Maximum used memory**
1. BIOSMILE	20 s	60 ms	1092 MB
2. CBIOSMILE	137 s	71 ms	1092 MB
3. RCBIOSMILE	25.4 s	43 ms	102 MB

## Discussion

### Advantages of (R) CBIOSMILE over BIOSMILE

CBIOSMILE and RCBIOSMILE excel at correcting two error types, (1) duplicate arguments and (2) overlapping arguments. An example of a duplicate argument would be:

*Partial amino acid sequences obtained from purified EBF were used to isolate* [*cDNA clones*_ARG0_], [*which*_R-ARG0_] [*by multiple criteria*_ARGM-MNR_] [*encode*_Verb Predicate_] [*EBF*_ARG1_]

Here BIOSMILE, working node by node, labels both “cDNA clones” and “multiple criteria” as ARG0—the former because it appears before “which”, and the latter because is the nearest noun phrase to the verb predicate “encode”. (R) CBIOSMILE avoids this error because the tree collective formulae limit the maximum number of any core argument type to one. Therefore, it labels only the node with the highest likelihood of being ARG0.

Similarly, (R) CBIOSMILE can avoid overlapping errors such as:

*Isolation of* [*a rel-related human cDNA*_ARG0_] [*that*_R-ARG0_] [*potentially*_ARGM-MNR_] [*encodes*_Verb Predicate_] [*the 65-kD subunit of NF-kappa B*] *(published erratum appears in Science 1991 Oct 4; 254) 5028 (: 11)*

In this example, BIOSMILE incorrectly labels the two overlapping nodes “*Isolation of a rel-related human cDNA*” and “*a rel-related human cDNA*” as ARG0. (R) CBIOSMILE does not make such errors because path collective formulae assert that only one node in a path of a parse tree can be an argument.

### Advantages of RCBIOSMILE over CBIOSMILE

According to the results of individual arguments for all argument types (shown in Table 8), we can see that RCBIOSMILE significantly outperforms CBIOSMILE in ARGM-ADV, ARGM-TMP, and R-ARG0. This may be because RCBIOSMILE employs several candidate identifiers to enhance the likelihood of true arguments being correctly labeled. Take the following sentence for example:

**Table 8:** Argument-wide SRL performance for each argument type

**Type**	**Description**	**BIOSMILE**	**CBIOSMILE**	**RCBIOSMILE**
**ARG0**	Main arguments whose definitions depend on the corresponding predicatecp	90.20%	91.85%	92.24%
**ARG1**		88.43%	89.89%	90.07%
**ARG2**		82.85%	83.00%	82.90%
**ARGM-ADV**	Adverbials, these are used for syntactic elements which clearly modify the event structure of the verb in question, but which do not fall under any of the argument types above	52.41%	51.24%	53.48%
**ARGM-DIS**	Discourse markers, these are markers which connect a sentence to a preceding sentence	78.51%	75.73%	73.68%
**ARGM-LOC**	Locative modifiers indicate where some action takes place	72.15%	71.99%	71.44%
**ARGM-MNR**	Manner adverbs specify how an action is performed	78.09%	79.18%	79.54%
**ARGM-MOD**	Modals are: will, may, can, must, shall, might, should, could, would. Phrasal modals such as "going (to)", "have (to)" and "used (to)" are also included	96.21%	96.63%	97.28%
**ARGM-NEG**	Negation, this tag is used for elements such as "not", "n't", "never", "no longer" and other markers	96.65%	95.98%	96.52%
**ARGM-TMP**	Temporal markers show when an action took place	56.52%	56.30%	57.99%
**Overall**		84.14%	85.16%	85.32%

[*Although lymphokine genes are coordinately regulated upon antigen stimulation*_ARGM-ADV_], [*they*_ARG1_] *are* [*regulated*_Verb Predicate_] [*by the mechanisms common to all as well as those which are unique to each gene*_ARG0_] .

The parse-tree-based candidate identifier employed by RCBIOSMILE recognizes the node whose span is “Although lymphokine genes are coordinately regulated upon antigen stimulation” as a candidate of ARGM-ADV or ARGM-TMP, and RCBIOSMILE adds the predicates “*candidate_role*(*p*, *i*, ARGM-ADV)” and “*candidate_role*(*p*, *i*, ARGM-TMP)” into the inference system. Therefore, this node can be successfully labeled as ARGM-ADV. Because CBIOSMILE does not use the candidate identifier, it has difficulty labeling such long-span nodes with the correct argument types.

### Disadvantages of (R) CBIOSMILE

RCBIOSMILE and CBIOSMILE, which use collective learning, outperform BIOSMILE in most argument types. Surprisingly, they perform worse than BIOSMILE in ARGM-DIS and ARGM-ADV. We believe that this may be because ARGM-DIS can be easily recognized by position information (ARGM-DIS usually appears at the beginning of a sentence), and duplication and overlapping errors (which collective approaches excel at handling) seldom occur in ARGM-ADV.

### Pruning errors of RCBIOSMILE

We observed that RCBIOSMILE cannot recognize some arguments that can be recognized by BIOSMILE and CBIOSMILE. After analysis, we found that such errors are caused by RCBIOSMILE’s pattern-based pruning step, as in the following sentence:

*However, the profound T cell deficit of nude mice indicates that* [*the thymus*_ARG0_] *is by far the most potent site for* [*inducing*_Verb Predicate_] [*the expansion*_ARG1_] [*per se*_ARGM-MNR_]*, even if other sites can induce some response acquisition.*

In this example, since the syntactic path from the node whose span corresponds to “the thymus” does not link to an ARG0 node and a verb-predicate node, this node is pruned. Therefore, RCBIOSMILE predicts a nearer noun phrase “the most potent site” as ARG0.

### Related work

#### Biomedical semantic role labeling corpus

PASBio [26] is the first PAS standard used in the biomedical field, but it does not provide a SRL corpus. GREC [27] is an information extraction corpus focusing on gene regulation events. However, GREC does not support the Treebank format SRL annotations [28]. BioProp is the only corpus that provides SRL annotations and annotates semantic role labels on syntactic trees. It is created by [24]. BioProp selects 30 most frequent or significant verbs found in biomedical literatures, and defines the standard of the biomedical PAS. Furthermore, in accordance with the style of PropBank [29], which annotates PAS on Penn Treebank (PTB) [28], BioProp annotates their PAS on the GENIA TreeBank(GTB) beta version [30]. GTB includes a collection of 500 MEDLINE abstracts selected from the search results with the following keywords: human, blood cells, and transcription factors and contains a TreeBank that follows the style of Penn Treebank.

#### Biomedical semantic role labeling system

Most semantic role labeling systems follow the pipeline method, which includes *predicate identification*, *argument identification* and *argument classification*. However, in recent years, instead of using the pipeline method, several researches have shown that using the collective learning method can outperform the traditional pipeline method. Riedel et al. [11] uses Markov Logic to collectively learn these stages on SRL. However, to the best of our knowledge, there seems to be no existing SRL system using MLN in the biomedical field. Dahlmeier et al. [31] uses the domain adaption approaches to improve SRL in biomedical field. Bethard et al. [32] considers SRL as a token-by-token labeling problem and focuses on the SRL in transport proteins. BIOSMILE is a biomedical SRL system that focuses on 30 frequently appearing or important verbs in biomedical literatures and trained on the BioProp, and it is based on the Maximum Entropy (ME) Model.

## Conclusions

Currently, a major problem of BioSRL is that most systems label every node in a full parse tree independently; however, some nodes always exhibit dependency. In general SRL, collective approaches based on the Markov logic network (MLN) model have been successful in dealing with this problem. In this paper, we explore the collective approach to BioSRL by building an MLN-based system.

Despite the convenience of modeling dependencies and the high accuracy of MLN, we have observed that it requires more memory and longer training times on a large corpus. This is an obstacle to applying MLN to BioSRL, which requires a large amount of training data to cover the wide variety of specialized biomedical subdomains. To reduce resource usage, we designed a pattern-based method to prune parse-tree nodes that may not have semantic roles. This method is applied to the parse trees in BioProp. To minimize the efforts of domain experts in manual pattern compilation, we developed an automatic pattern generation approach. The pruned annotated parse trees are used to train a resource-saving MLN-based system, which is referred to as resource-saving collective BIOSMILE (RCBIOSMILE).

Our experimental results show that our proposed CBIOSMILE system outperforms BIOSMILE, which is the top BioSRL system. Furthermore, our proposed RCBIOSMILE maintains the same level of accuracy as CBIOSMILE using 92% less memory and 57% less training time. This greatly improved efficiency makes RCBIOSMILE potentially suitable for training on much larger BioSRL corpora over more biomedical domains. Compared to real-world biomedical corpora, BioProp is relatively small, containing only 445 MEDLINE abstracts and 30 event triggers. It is not large enough for practical applications, such as pathway construction. We consider it of primary importance to pursue SRL training on large corpora in the future.

## Competing interests

The authors have no competing interests.

## Authors' contributions

RTH Tsai designed the algorithm and all the experiments, wrote most of this paper, and guided the whole project. PT Lai wrote the programs and conducted all experiments. All authors read and approved the final manuscript.
